# Patient perspectives on non-response to psychotherapy for borderline personality disorder: a qualitative study

**DOI:** 10.1186/s40479-023-00219-y

**Published:** 2023-04-18

**Authors:** Jane Woodbridge, Michelle L. Townsend, Samantha L. Reis, Brin F. S. Grenyer

**Affiliations:** Illawarra Health and Medical Research Institute and School of Psychology, University of Wollongong, Building 22, Wollongong, NSW 2522 Australia

**Keywords:** Borderline personality disorder, Non-response to psychotherapy, Treatment failure, Safe therapeutic alliance

## Abstract

**Background:**

Despite increasing evidence for the effectiveness of psychotherapy for Borderline Personality Disorder (BPD), estimates show that approximately half of those in treatment do not clinically improve or reach reliable change criteria. There are limited qualitative descriptions of treatment factors associated with non-response from the perspectives of those struggling to improve.

**Method:**

Eighteen people (72.2% female, mean age 29.4 (*SD* = 8)) with experience of receiving psychotherapeutic treatment for BPD were interviewed to obtain their perspectives on hindering factors in treatment and what may be helpful to reduce non-response. The data in this qualitative study was analysed thematically.

**Results:**

Four domains were created from the insights patients shared on non-response and what may be needed to mitigate it. The focus of Domain 1 was that therapy cannot be effective until two factors are in place. First, the patient needs sufficient safety and stability in their environment in order to face the challenges of therapy. Second, they need to be able to access therapy. Domain 2 described factors the patients themselves contribute. The themes in this domain were described as phases that need to be progressed through before therapy can be effective. These phases were ceasing denial that help is warranted and deserved, taking responsibility for behaviours that contribute to unwellness, and committing to the hard work that is required for change. Domain 3 described how the lack of a safe alliance and ruptures in the safety of the relationship with the therapist can contribute to non-response. Domain 4 was comprised of factors that patients identified as supportive of moving through the barriers to response. The first theme in this domain was prioritising the safety of the therapy relationship. The second theme was giving a clear diagnosis and taking a collaborative approach in sessions. The final theme described the importance of focusing on practical goals with the patient to create tangible life changes.

**Conclusion:**

This study found that non-response is complex and multifaceted. First, it is clear that systems need to be in place to support access to adequate care and foster life stability. Second, considerable effort may be needed at the engagement phase of therapy to clarify expectations. Third, attention to specific interpersonal challenges between patients and therapists is an important focus. Finally, structured work to improve relationships and vocational outcomes is indicated.

**Supplementary Information:**

The online version contains supplementary material available at 10.1186/s40479-023-00219-y.

## Introduction

The evidence-base of effective treatments for BPD has continued to be amassed over the last four decades. Numerous outcome studies testing the effectiveness of psychotherapies specialised for BPD have demonstrated their ability to decrease BPD symptoms and other relevant clinical features such as self-harming and suicidal behaviours, substance misuse, service use and hospital admissions [ 8, 14, 26, 28, 30, 44, 49, 55, 82]. However, a large proportion of people remain non-responsive to psychotherapy for BPD. A recent systematic review reported that approximately half of the patients included did not achieve a reliable reduction in BPD symptoms after receiving psychotherapy [92]. Published treatment outcome studies report that between 6% to 81.3% (*M* = 45%) of patients do not respond (as defined by a lack of BPD symptom reduction) to some of the main treatments available for BPD, i.e., Dialectical Behaviour Therapy, Schema Focused Therapy, Transference Focused Therapy, Cognitive Analytic Therapy and Mentalisation Based Therapy [ 27, 28, 30, 35, 44, 47, 49, 50, 63, 72]. Furthermore epidemiological, naturalistic and treatment outcome studies all report that the majority of people with BPD experience difficulties achieving healthy levels of social or occupational functioning and continue to be high frequency service users [ 2, 4, 20, 22, 25, 81, 83, 94].

Although the problem of non-response is not new, available research has provided contradictory findings that are primarily focused on patient-related factors. For instance, emotional instability, emptiness, dissociation, self-harm and suicidality have all been associated with both negative and positive therapeutic outcomes [13, 15, 36, 48, 60, 75, 88, 93]. A systematic review of predictors of treatment response (as defined by symptom reduction) found no consistent relationship between outcomes and sociodemographic variables, pre-treatment comorbidity or psychotropic medication use [7]. Therefore, there is still much to learn about patient-related factors and how they may contribute to the problem of non-response.

Other authors have sought to explore the treatment of personality disorder at the system-level and found that accessibility was a problem. For example, [18] found that there was a lack of available treatments, especially in regional areas. Those areas that did have specialist treatments for BPD tended to have long waiting lists, and the subsidising initiatives did not cover the frequency or intensity of treatment required to recover from BPD.

Treatment guidelines state that the recommended treatment for people with BPD is a sufficient dose of evidenced-based psychotherapy in the community [66, 71, 77]. It may be important to seek the perspectives of, and collaborate with, people with a lived experience of receiving psychotherapy for BPD to understand response and non-response [6, 77]. Qualitative research approaches may offer unique and novel perspectives on this issue.

Past qualitative research into the experience of recovery from BPD has revealed that the therapeutic goals of the service can be misaligned with the therapeutic goals of the individual, which are often more focused on improving function [45, 64]. A narrow focus on symptom reduction and lack of focus on wider aspects of recovery such as support to gain stability in relationships, living conditions and work could be contributing to non-response [39].

Although no direct research into non-response to psychotherapy for BPD was able to be located, a meta-synthesis of qualitative studies on the perspectives of individuals with BPD on treatment experiences and recovery processes, reported some unhelpful treatment characteristics [46]. Patients found it unhelpful when their clinicians were experienced to be judgemental, distant or not understanding. Patients were subsequently left feeling undeserving of support, criticised and isolated. When clinicians were perceived to respond only to risk of harm problems, patients felt that their distress was not addressed. Not feeling like an equal partner in treatment by having goals imposed upon them created a perception that therapy was too rigid and left patients feeling angry, powerless and unmotivated to take action towards progress. Patients also reported that open ended therapy, where the clinician was not pushing for change or offering any clear solutions to problems, was confusing and unhelpful.

There is evidence that there exist factors which may contribute to non-response to psychotherapy for BPD. However, these factors are yet to be investigated thoroughly or directly. Qualitative studies suggest that differing definitions of recovery, misalignment of treatment goals, mis-attuned therapeutic alliances, non-collaborative treatment without a focus on solutions or change are all factors that may contribute to non-response. Non-response is infrequently investigated, and therefore, remains poorly understood. More research is needed to explore and understand the problem. The present study explores the insights of patients with a lived experience of receiving psychotherapy for BPD, with the aim of understanding treatment non-response from the patient perspective.

## Method

### Participants and data collection

Patients with BPD who were part of a longitudinal study of stepped care treatment [38, 42], were invited to participate in an interview about non-response and what may be required to address it. All gave written informed consent following Institutional Review Board approval. Demographic and clinical care details are reported in Table 1. The qualitative interview was audio recorded and transcribed verbatim. The first question was *‘We know that most people respond well to psychotherapy. Research also tells us that up to one half of people who receive psychotherapy for BPD don’t respond well. Why do you think that is?’* That question was then followed with more specific prompts to enable further exploration into patients’ perspectives on what makes psychotherapy and psychotherapists ineffective. (Interview Questions can be found in Additional file 1). Interviews took 45 min on average.

**Table 1:** Demographics, diagnosis and treatment information

	N (%)	M (SD)	Range
Total N	18		
Non-Binary People	2 (11.1)		
Females	13 (72.2)		
Males	3 (16.7)		
Age		29.4 (8.0)	19—46
Occupation			
Full Time Employment	4 (22.2)		
Part Time Employment	6 (33.3)		
Unemployment Benefit	3 (16.7)		
Student Allowance	2 (11.1)		
Pension	3 (16.7)		
Currently studying	18 (100)		
Currently in a relationship	9 (50)		
Currently taking psychotropic medication	8 (44.4)		
Currently in treatment	12 (66.7)		
Primary Therapist			
Psychologist	11 (61.1)		
Counsellor	1 (5.6)		
Social Worker	1 (5.6)		
No longer in treatment	5 (27.8)		
Months in treatment with current therapist		15.3 (9.7)	2—36
Number of therapists engaged with			
1—3	8 (44.4)		
4—7	5 (27.8)		
8—11	4 (22.2)		
12—19	1 (5.6)		
No. BPD criteria currently endorsed (out of 9)		5.6 (2.7)	0—9
No. BPD lifetime criteria (out of 9)		7.5 (1.5)	5—9
Patients currently meeting criteria for BPD	17 (94.4)		
Patients who previously met criteria for BPD	18 (100)		
Lifetime comorbid diagnoses			
Depression	17 (94.4)		
Anxiety	16 (88.9)		
Obsessive Compulsive Disorder	2 (11.1)		
Bipolar Disorder	7 (38.9)		
Phobias	3 (16.7)		
Attention Deficit-Hyperactivity Disorder	3 (16.7)		
Post Traumatic Stress Disorder	10 (55.6)		
Complex Post Traumatic Stress Disorder	6 (33.3)		
Eating Disorder	2 (11.1)		

### Data analysis

Data were analysed using [16, 17] six phased approach to reflexive thematic analysis. The stages were moved through recursively. Data familiarisation commenced at data collection stage as the main researcher conducted the interviews. Data familiarisation continued through reading and re-reading the interview transcripts by two coders (JW and MT). Notes based on meaning were written in the margins and any data extracts that were recurring and meaningful were highlighted. Semantic and latent coding took place through two coding sweeps across the entire data set. Codes were grouped into nodes using NVivo 11, a software program for qualitative analysis. Themes were constructed deductively from nodes that shared similarly patterned meanings.

Refinement was conducted by assessing each theme for internal homogeneity and external heterogeneity. Visual maps were used to make sense of relationships between the themes and the overarching research question. The resulting themes were grouped into semi-sequential domains and named based on their central unifying concept. To increase the credibility of the findings, some measures suggested by Noble and Smith [67] were taken. Themes were validated and evidenced through peer debriefing and frequent discussion between all authors, who also engaged in self-reflexivity to reduce possible biases. As a further measure to reduce bias two other experts, the first authors’ primary and secondary clinical supervisors, who were not involved in the project were consulted. Both have doctorates and over 20 years’ experience working with personality disorder in private and community mental health roles. Lastly, rich and thick verbatim descriptions of participants’ insights were included in the results to allow the reader to judge whether themes were true to participant accounts.

## Results

When creating a subgroup to select from within the larger cohort, certain characteristics were considered including age, gender, history of treatment and exposure to different therapy models. This was to ensure a sufficiently diverse set of perspectives and heterogeneity of the final sample. Patients were also entered into the subgroup based on length of time since last interview to ensure the research was not burdensome. From this subgroup, patients were randomly selected for invitation to participate in the interviews. Data saturation (i.e. no new themes emerged) was reached with 18 participants. The number of therapists patients had engaged with varied. Almost half (44.4%) of the patients had engaged with one to three therapists, while one patient had 19 therapists across her treatment history. The sample ranged in age from 19 to 46, there was a mix of genders and there were differences in vocational and employment characteristics. All patients met lifetime criteria for BPD and 17 (94.4%) currently met criteria. There were no significant differences in responding (according to number of currently endorsed criteria) by clinical or demographic grouping variables, (gender, employment status, psychotropic medication use) nor was age, length of time in treatment or number of therapists engaged with correlated with number of BPD criteria currently endorsed. All clinical and demographic data can be seen in Table 1. At the time of the interview, 12 of the 18 (66%) patients felt they were responding well to treatment despite current challenges, and four felt they had not yet reached a recovery phase. There were no demographic or clinical differences between these two groups, nor was there a significant difference in the number of currently endorsed BPD criteria between the groups, and so all were analysed as a single sample as representing a range of views.

**Fig. 1:**
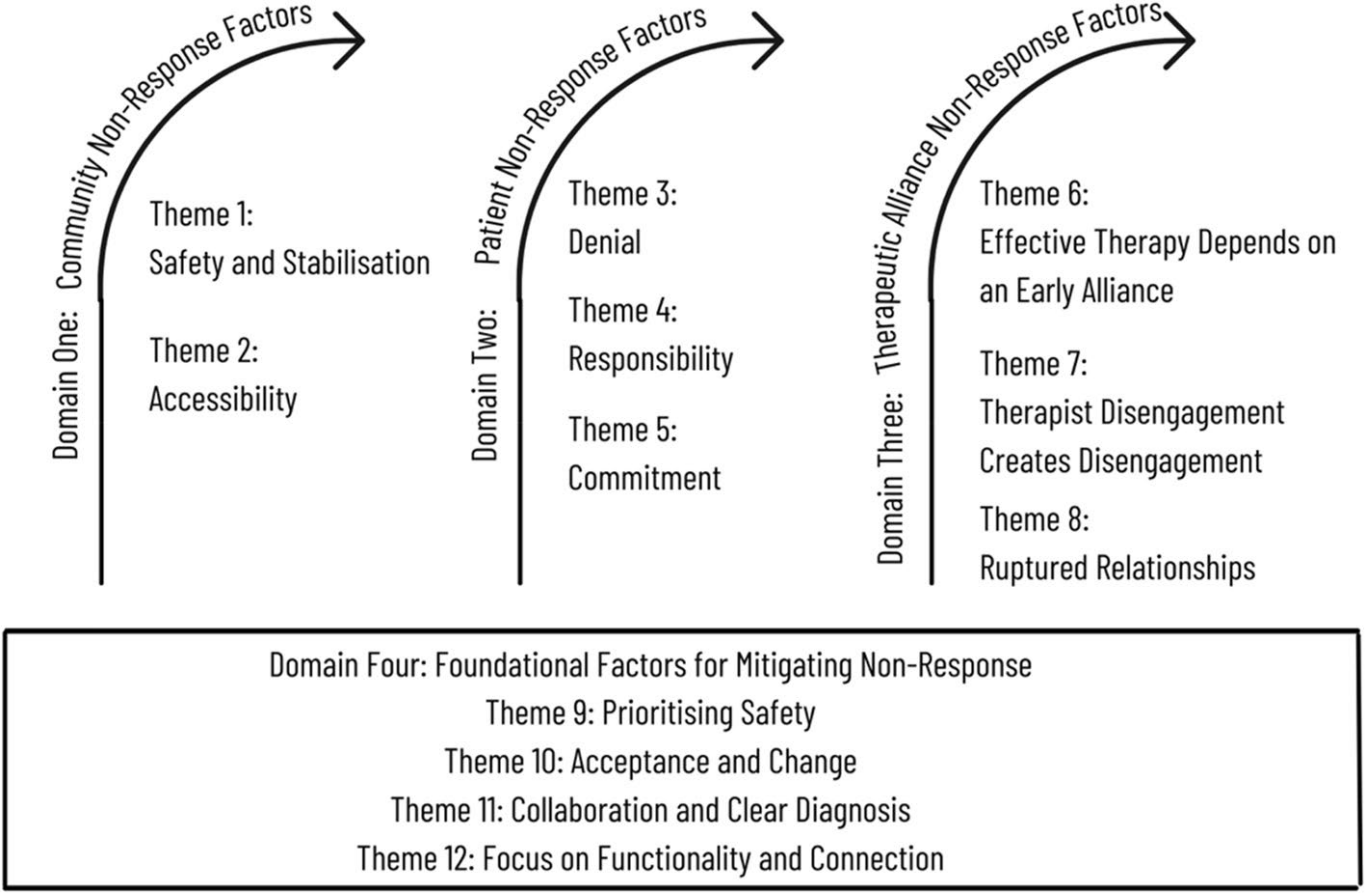
Diagram of relationship between domains

Four broad overarching domains consisting of twelve themes were constructed from the insights patients shared on non-response to psychotherapy for BPD. Domains and themes, and the relationship between the domains are depicted in Fig. 1. The first three Domains are depicted as sequential phases that the patient needs to move through before response to therapy can occur. The themes that compose the first three Domains are created from the patient-identified barriers to response. The themes that compose Domain 4 are created from the patient-identified factors that, if present, would support patients to work through the barriers in the first three Domains.

### Domain 1: Community non-response factors

Domain 1 captured the common recognition among patients that certain broader environmental, social, and service provider pre-conditions must first be met before psychotherapy can commence and be effective.

#### Theme 1: Safety and stabilisation

‘Therapy only works if you are safe, stable and supported’.

Theme 1 was generated from discussions regarding the necessity of having stability across multiple life domains before one can engage consistently in therapy. The required factors were to be free from living in active trauma, to have a safe and stable environment with minimal chaos and a supportive social network. Patient – *‘…where before I was in this chaotic position, I was really struggling in a lot of aspects of my life. Very – you know, struggling to maintain jobs and really struggling with living circumstances and, particular hard life events that were happening. Whereas I’m in a much better position now. If something bad does happen I can cope with it because I’m settled. I’m not in the constant chaotic mess.’* Patients also described the necessity of having people in their lives that understood the validity of mental health problems and supported them to get professional help, instead of holding stigmatised attitudes about mental illness. Encountering unhelpful attitudes in patient’s social environments were described as shame inducing, which acted as a barrier for them to be able to seek and engage consistently in therapy. Patients explained that without first reaching a level of safety and stability in their external environments, they could not reach a level of psychological stability and safety required to truly engage in and benefit from therapy. Patient – *‘Like I feel like the person really needs to be in a safe place for therapy to kind of work effectively, because it is requiring so much vulnerability, it’s hard to imagine someone whose been living in active trauma to like, get benefit from something like talk therapy, unless they’re obviously being empowered to leave that abusive situation. But it’s just like, I feel like the vulnerability required for it to be super effective and efficient and really life-changing requires like a foundation of some kind of safety for the person so that they can kind of, you know, undress and strip down and figure out what’s going on.’*

#### Theme 2: Accessibility

‘Therapy only works if you can get it’.

Patients noted that for therapy to be effective you must first be able to access it. While patients discussed the lack of information available regarding how to access therapy, the main concern was the lack of therapists available. Patients described encountering long waiting lists and long periods between appointments as barriers to engagement. Some patients reported services to be so overburdened that consistent appointments with the same therapist were often unobtainable. Patients viewed this situation as unacceptable, especially when people experience acute crises and need immediate and frequent care. Patient – *‘… especially when people – I know that people who suffer from this borderline personality or people who are, um in this intense kind of crisis mindset, it’s really immediate attention that they need, with regular appointments, and I know that I’ve gone to my doctor at times and said, “I am here because I don’t know what to do. My appointment is not for another two months because I couldn’t get in and I really need to talk to someone now, I need to do something now.’* Patients also expressed exasperation about discussing past trauma in session, only to be left alone with open emotional wounds because the next available appointment was not for many weeks. Another frequently cited barrier to obtaining therapy was affordability. It was consistently recognised that the public healthcare system may be inaccessible due to being overburdened, while private psychologists, who also have long wait lists, are too expensive even after accounting for government subsidies. Patient – ‘…* there’s just not enough, you know, spaces, appointments, especially in the public health system. And Medicare [governmental subsidies for private psychological treatment] doesn’t cover the full fee. I had – I had to cancel appointments when I was younger because I just couldn’t afford it at the time.’*

### Domain 2: Patient non-response factors

Domain 2 was informed by retrospective insights about what patients considered they were doing to contribute to their non-response.

#### Theme 3: Denial

‘Therapy didn’t work when I was stuck in denial that I needed and deserved help’.

This theme is drawn from the recognition of patients that, in hindsight, being stuck in denial had impeded their ability to effectively engage in therapy. Patients described how in the past they were in denial about how unacceptable their living situation was, how toxic or abusive their relationships were, and how poor their mental health was. Patient – *‘… I found that there was a few things that I was lying to myself about. And not admitting to how badly some things were affecting me. Some external factors, like how people treated me, and how much I actually took that on myself, and how much that played on my schemas of feeling inferior and feeling worthless. Because I would tell myself that, you know, that’s, that’s just how it is, I’m used to it now. It’s okay.’* Patients described this as a stage of denial that had to be progressed through before they could take the initial step of admitting that there was a problem and that they needed help. Patient – *‘So obviously it sunk me down into more darkness first, but I think you have to hit rock bottom before you can be out of that denial stage, it needs to kind of hit you like a tonne of bricks.’* Furthermore, patients noted that progressing through their denial phase was contingent on developing enough self-worth and the resolve that they deserved for things to be different. Patient – *‘ I just think that I needed to realise that I did matter because I didn't really see myself as an important or that it would make a difference whether I was here or not.’* Patients acknowledged the need to be brave enough to embrace the vulnerability that came with letting go of defences, of letting the therapist in, and of baring the whole truth, so the therapist could provide suitable help. Patient – *‘So I was dishonest in therapy and I was kind of like beating around the bush trying to get help without being completely honest, because I was scared of being honest.’*

#### Theme 4: Responsibility

‘Therapy didn’t work when I was unwilling to accept responsibility for my role in contributing to my problems’.

Patients understood that after they resolved their denial, and accepted they needed help, they also needed to accept that that their behaviours were perpetuating their mental health problems. Patient – *‘… possibly not willing to look intensely at their own behaviours that sort of contribute to the issues they may be having in their life… and kind of scrutinising your behaviours and your thought processes’.* Patients acknowledged that this process can be very painful, yet necessary. Patient – *‘Oh, like, well, it’s never going to be comfortable to admit that maybe you’re doing something wrong or that maybe you’re the problem in situations.’* This process was said to help patients move past using therapy solely as a space to vent (pour out problems without taking action to resolve them). Patient – *‘Because if you’re just going to go into your sessions, and just want to whinge and moan about how hard your life is, and how the world’s against you, and how horrible things are, then if you’re not delving into the actual reasons why you feel that way, then I just don’t think you've got any, any platform to work on.’* Patients spoke of the importance of no longer using therapy to vent, and instead as a place to take full accountability, which requires an ability to tolerate high levels of discomfort. Patient* – ‘Like, if you can’t take responsibility for or understand that your experience in the world is all based on perspective, as opposed to it all just happening to you, I think that that’s when people might find it really tricky.’* Acceptance of responsibility was described as letting go of old coping strategies that were no longer functional. Patient –* ‘Um, I think I just reached a point where I became very aware that the coping mechanisms that I had developed from a very young age were no longer serving me, or benefiting me in any way. I had to become very willing to let go of that and learn new strategies, but that can almost be quite difficult in itself because it’s like these defence mechanisms and coping strategies, they’re almost like a dysfunctional old close friend in a way.’*

#### Theme 5: Commitment

‘Therapy didn’t work when I was unwilling to do the hard work, inside and outside of sessions, required for change’.

Patients recognised that they must accept that they need help, then accept that they are contributing to their problems, and finally that they need to commit to exerting consistent effort inside and outside of sessions to make lasting change. The recognition that they had some power to change was described as the first step. Patient – *‘… instead of sitting there just freaking out. Like I said, it’s hard to get out of that freak-out stage but there needs to be something like a trigger there that goes, ‘hey, hang on a second, you can do something about this.’’* This was followed by the realisation that change, although hard, is the responsibility of the patient. Patient – *‘I feel, actively choosing is probably the best way, because look, I’ve been in a place where you feel like you can’t, and you don’t want to, like, it’s all too hard and you think you’ve got no one else can help you and, well I can’t – it comes down to yourself’.* Patients described how the realisation of how much continual work they had to do as overwhelming, but it was exactly what was required. Patient – *‘but you know, their victim mentality can sort of come into play a bit; not wanting to take responsibility for the effort that they have to put in because therapy is more than just talking for an hour once a week or once a fortnight. It’s a lot of work that consumes every day.’*

### Domain 3: Therapeutic alliance non-response factors

Domain 3 was created from the insights patients shared about a perceived lack of safety in the therapeutic alliance, and what therapists do to contribute to non-response.

#### Theme 6: Effective therapy depends on an early alliance

‘Therapy only works if you click with your therapist’.

The majority of patients asserted that the effectiveness of psychotherapy was contingent on an immediate, safe and secure therapeutic alliance. Patient – *‘I think one of the biggest and most important things is having a therapist that you feel safe with and you can trust and you just click with.’* Patients used many words including ‘click’, ‘vibe’, gel’, ‘symbiosis’, ‘connection’, ‘bond’, to discuss the therapeutic alliance. Although the click was a difficult phenomenon to articulate, and patients were at times unsure of what *‘it*’ was, all patients were confidently sure of whether it was or was not present, and of its profound importance. Patient – *‘ I imagine that that gel is what a lot of people would talk about because it's very there or not there. And — and yet it’s not tangible.’* Patients felt that the click happened almost immediately, instinctively and below conscious awareness. Patient – *‘… you can pick it when you start interacting with someone if you are down with them. I think it’s just a human instinct. Like, an instinctual thing.’* Patient—*Oh it’s definitely not conscious*’.

Patients noted that you could not force the ‘click’; it was reported to either happen naturally or not at all. Patient – *‘…it's just like the connection that you feel, like a natural connection.’* Patient – *‘I think it’s like falling in love with someone, right? Not that you fall in love with them. But when you’ve got chemistry with someone — you either know if it’s there and you can build on that or you don’t …’* Accordingly, the patients’ advice was invariably to keep trying different therapists until one was found that could be ‘clicked’ with. Patient – *‘They’d just have to keep trying different people until they connect with someone.’* There was also a recognition that searching for a therapist you can connect with is a difficult and costly task. Patient – *‘…the problem itself … that we’re talking about at the moment is an extremely hard one to solve without trial and error. So from a 10-session perspective, unless you get that connection you’re going to lose 20% of your, um, healthcare plan [governmental subsidies for private psychological treatment]’*.

#### Theme 7: Therapist disengagement creates disengagement

‘Therapy doesn’t work when therapists don’t genuinely care about me’.

This theme was created from patient observations of therapist behaviours that indicated a lack of motivation and investment in the therapeutic relationship or genuine care in the patient and their recovery. Patient – *‘And it just kind of felt like they were going through a workbook, essentially, like, I’ve got to do step one and then step two…’* Patients asserted that therapy does not work when therapists did not listen or take their concerns seriously, then sent them home with handouts or advised that meditation apps would make things better. Other therapist behaviours perceived to indicate disengagement and lack of care were not maintaining common courtesies and not holding the therapeutic frame, i.e., being late, answering calls and typing in session. Patient – *‘… it felt dismissive because she would just keep giving me paperwork on breathing techniques and, like, here’s some recent studies on anxiety and depression. Go home and study up about your own mental health. And her phone would be going off and she’d be like, ‘Oh sorry, it’s just my daughter’. And she’d be typing on the laptop as I was talking, asking me to repeat things.’* Inattention, lack of genuine care, and the sense of being dismissed lead to patients also disengaging from therapy. Patient – ‘*… like, looking out the window or their phone. I’m, like, well, I’m here, spilling out my guts and they’re not paying full attention to me so why would I bother’.*

#### Theme 8: Ruptured relationships

‘Therapy doesn’t work when therapists make me feel unsafe’.

This theme is created from patient’s perspectives on the behaviours therapists can exhibit to rupture a safe therapeutic alliance, which in turn, contribute to non-response. One of the frequently reported, highly valued elements of psychotherapy was being able to speak freely without needing to worry about judgement, consequences or the emotional impact on the listener. Maintaining the safety for patients to openly express themselves appeared to be delicate – even subtle misattunements and communication mistakes were described as enough to fracture the safety of the holding environment. When therapists were seen to break confidentiality, anonymity or neutrality by penetrating this space with their own displays of emotion or personal opinions, it was experienced as intrusive and was reported to influence patients to become defensive and careful with what they spoke about. Patient – *‘…rather than let me voice it… she had a very big personality that she brought to the room, and it was very domineering, So I did struggle to open up to her. Because I was just cautious of how I am going to word this so that she lets me finish my sentence. And I think that’s counterproductive. I just don’t think that you should have to worry about your wording and things like that’.* This type of therapist behaviour was often cited to be what lead to patients terminating therapy. Patient – *‘I feel like you’re judging my life choices and my morals… I don’t subscribe to a particular religion, and they definitely did. Like, there are a few comments made, like “If you’re a feminist, you’re not going to like what I have to say next”… I could have been really quite sensitive about it. But it was enough to… I just didn’t book another appointment and I’d been talking to that person for five years.’*

Patients discussed therapist behaviours that were experienced as highly distressing, such as being condescending, threatening and shaming. These overt displays of unmanaged countertransference contaminated the safety of the holding environment, breached the patient’s sense of trust, and essentially prevented therapy from being effective. Patient – *‘And so she threatened ‘If you come back in here next week with fresh harm scars, I’m going to phone the ambulance and they’re going to come and they’re going to pick you up in front of all of these people in the waiting room and they’re going to take you to the hospital’ and I never went back there again.’*

### Domain 4: Foundational factors for mitigating non-response

Domain 4 is comprised of the insights patients shared about which factors to focus on as a way to address non-response.

#### Theme 9: Prioritising safety

‘Therapy could work better if the therapist prioritised helping me feel comfortable and accepted’.

This theme emphasises the importance of a safe relationship as the foundation of effective psychotherapy. Patients described a therapist’s ability to be attuned and responsive to the patient’s emotions as the predominant factor that supported the development of safety in the relationship. Patient – *‘She puts her mood to suit our mood. Like, she makes her mood, very calming, makes us feel comfortable… I can’t even explain it.’* Patients asserted that active listening, demonstrated by holding patients in mind, showed that the therapist was interested, invested and genuinely cared about the patient. Therapists who were warm, compassionate and non-judgemental supported patients to feel safe to express themselves freely and remain their authentic selves. Patient – *‘Well, I think one of the biggest and most important things is having a therapist that you feel safe with and you can trust and you just click with. You’re able to just interact with them well, not kind of have to change how you talk to suit their style of doing things. Being able to remain authentic.’* Patients identified that being available, reliable and relatable by demonstrating lived experience also facilitated the development of a safe therapeutic relationship. Patient – *‘Just in my own personal experience I was able to click and gravitate more towards those with lived experience.*’

#### Theme 10: Acceptance and change

‘Therapy could work better if the therapist understood and accepted that my behaviours and emotions can be a result of my situation, while still gently pushing me to change’.

This theme was created from the discussions around the importance of their behaviours and emotional responses being understood as originating from trauma or context. Patients wished for therapists to develop a nuanced and comprehensive understanding of each individual as unique, by knowing their specific circumstances and history. Patients believed that this would require therapists to let go of preconceived notions and assumptions based on patient’s diagnoses of BPD. Patient – *‘…generally there’s a bit of dismissal, there’s a shift in tone, a shift in decorum from the clinician. So yeah, I think a little bit more flexibility and working with a person, rather than working with a diagnosis.’* Patients asserted that this holistic understanding of a person’s behaviours and emotional responses in context helped the therapist to provide genuine validation, and that receiving such validation was highly valued. Patient – *‘I found it really useful when my therapist put himself in my shoes, and sat back was like, ‘Wow, no wonder you’re as low as you are.’’* Patients emphasised that contextual understanding of patients is also essential for the delivery of effective therapy. Such knowledge was seen to allow for the therapy to be adjusted to individual needs and to move responsively between supportive and expressive stances as needed. Patient* –* ‘*I think it’s important in a way for the patient to get challenged at times. And I think it has to be gentle, you know, that kind of push for change and trying to encourage a perspective shift. It definitely has to be gentle.’*

#### Theme 11: Collaboration and clear diagnosis

‘Therapy could work better if the therapist gave me a clear diagnosis and worked with me to help me get better’.

This theme is created from the perspective shared among patients that they highly valued therapists who were transparent, provided rationales for interventions and offered them choices and opportunities to have input into their own treatment. This demonstrated respect, equality and nurtured the development of shared goals that could be worked towards as a team. Patient – *‘The most important one to me that I think worked was feeling like it was a team effort. Like I wasn’t going to therapy and receiving help, I was going to therapy and working through my problems with the therapist’.* A collaborative approach was described to empower the patient to gain control over their own life. Patient – ‘*To have that open discussion, to let me be part of my own treatment path, as opposed to just saying ‘This is what we’re going to do, this is how we’re going to tackle it’. For it to be an actual discussion so that I feel like I have some control. Because essentially, it is my life.’* Patients also described how being supported to develop a more sophisticated understanding of their internal world was the foundation for change. Patient – *‘I think for me, my first real indication that I was on that path was just a bit more insight. Say I’d act in like a certain way and then I’d think to myself, ‘Okay, like, that’s old behaviour, that’s not how I want to engage anymore’. So, it really started with heightening awareness.’* Lastly patients discussed the necessity of receiving a stable diagnosis of personality disorder because being given many varied and inconstant diagnoses was frustrating and confusing. Many patients described how being given a well-considered diagnosis can be liberating, and a relief to know it is a diagnosable condition that is treatable. Patient – *‘That diagnosis was really powerful for me, because it took a lot of self-blame away, because I wasn’t the screw-up. It wasn’t my fault that I responded to things abnormally. And it wasn’t until someone gave me a diagnosis and said, ‘It’s not your fault that you’re wrong—that you’re responding to these abnormally’. And when someone was able to say, ‘This is what’s wrong. And the thing is, we can now help you fix it.’’.*

#### Theme 12: Focus on functionality and connection

‘Therapy could work better if the therapy was practical and helped me make real life changes’.

This theme is built on the recognition that not only does therapy need to focus on the traditional therapeutic goals of symptom reduction, but it also needs to focus on supporting people to set practical and actionable goals that support progress towards social and occupational function. It was recognised that being encouraged to envision a better life facilitated such progress. Patient – ‘*I was in a place where I was able to see change. I was able to envision change. I was in a position where me and my therapist, we took small steps. We worked on getting my licence and we worked on getting me into university and then we kept working on getting a better job and things like that. And because I was able to envision things that I could do in the future, we – I was able to do so*.’ Patients discussed the value of being connected with social support via support groups, group therapy, or including chosen and important people in the therapy. Patient – *‘Because I feel like when you’re going through something like mental health you always feel alone. You think you’re crazy. You feel like you’re insane. But being exposed to other women going through the same thing, or who were also having mental health issues, was already healing in itself.’*

## Discussion

This study sought to understand non-response to psychotherapy for BPD from the perspective of people with experience of receiving psychotherapy for the disorder. Although most (66%) felt they were currently responding well to therapy, all continued to endorse moderate to high levels of BPD symptoms. This is consistent with previously reported perspectives of people with BPD, that although reducing symptoms is important, living a functional, meaningful and engaged life is more highly valued than symptom reduction alone [29, 52, 65].

Four domains were created from the insights that patients shared on non-response and how it may be reduced. The first three domains could be considered essentially sequential phases, while the fourth domain could be considered the foundational treatment factors that mitigate non-response. Domain 1 captured the acknowledgment that the wider community and health care accessibility pre-conditions must first be met before therapy can commence. Some of these conditions were external and are the shared responsibility of the patient, service providers, community and government. In theme one patients described the necessity of reaching an improved level of safety and stability in their environment before they could engage meaningfully in psychotherapy. Specifically, they needed their daily lives to be free from chaos and danger, and their social networks to be supportive of mental health treatment. This theme is congruent with the three-phased approach to trauma therapy in which phase one focuses on stabilising external and internal safety [23, 41], and with previous qualitative research in which people with BPD emphasised that developing a sense of environmental safety is the first phase of recovery [19]. In theme two patients identified that an initial factor implicated in non-response was the inability to access therapy, either by limited availability or prohibitive cost. This finding calls for more funding for mental health services and is consistent with previous authors who have identified the lack of treatments available, especially for people with BPD who require specialist treatments for longer durations and higher intensities [18, 43]. It is also noted that many patients had tried to engage in therapy with a high number of therapists. This could be reflective of a systemic problem in which it is difficult to be assigned to one consistent clinician in public services, which in turn creates a barrier to the establishment of a safe therapeutic alliance. Alternatively, it could demonstrate the episodic nature of treatment episodes along a longer history of seeking mental health care [37].

Domain 2 comprised insights about what patients considered they did to contribute to their own non-response. The themes in Domain 2 represent sequential phases of barriers patients described as necessary to resolve before therapy can become effective. Theme three captured the recognition that patients can often be in denial about their current level of dysfunction and that there is a need to accept that one’s mental health problems are severe enough to warrant help. Patients described how being closed to engagement, or refusal to embrace the vulnerability that comes with being honest with oneself and the therapist, can create barriers for the effectiveness of therapy. The development of self-worth was thought to facilitate the process of working through this denial phase. Theme four described the need for patients to accept their role in contributing to their own problems and the necessity of generating willingness to endure the emotional pain that comes with truthful self-reflection. Patients noted that this process can be painful, but it is required so that they can take responsibility for their own mental health. These themes are similar to a previous qualitative study about recovery from BPD in which patients described ‘being stuck’ in continued use of maladaptive coping methods and poor insight in the initial stage of recovery [65]. Theme five illustrated the common perception that therapy will not be effective until the patient is prepared to commit to the hard work required to make lasting change. Taken together these findings capture the concept of readiness, yet go deeper, to explain what readiness is. Domain 4 of the present study explains what patients need to become ready and echoes previous qualitative research on recovery from BPD which has also noted that the acceptance of the need for change, reflection on the patient’s own role in creating difficulties, and recognition that change requires constant hard work are precursors to recovery [29, 46, 65].

Domain 3 was created from the insights patients shared about how the lack of safety in the patient-therapist relationship was a major contributor to non-response. This finding reflects previous research that has reported that the development of a strong therapeutic alliance in the early phases of treatment is crucial for people being treated for personality disorder [7, 12], McMain et al. [59]. Theme six was built from the discussions about the therapeutic relationship. Patients described the phenomenon of the alliance, or ‘click’ as they termed it, as an instant felt state of trust that was difficult to articulate. [69] concept of neuroception could offer an explanation for the immediate ‘click’, or lack thereof. Neuroception is said to be the preconscious neurophysiological mechanism that humans use to detect cues of safety and danger. People with attachment difficulties can have faulty neuroception [69]. There is evidence that people with BPD have differences in their autonomic nervous system which influence them to be more likely to activate defensive physiological states in safe environments compared to controls [5, 87]. This could lead to misperceiving cues of danger from therapists, akin to mentalising impairments [10, 32, 76]. Faulty neuroception could also explain why most patients explained that non-response was due to the lack of an immediate alliance and why the sense of a safe relationship was so difficult to find and develop. Other explanations for the difficulty of establishing a safe working alliance could include insecure attachments [53, 54], their creation of epistemic mistrust and subsequent difficulties with mentalising [31–33]. Future research on the relationships between neuroception and development of alliance for individuals with BPD may shed light on current findings.

Patients identified a variety of unhelpful behaviours their therapists engaged in that ruptured the safety of the alliance. In theme seven patients described how therapist disengagement can contribute to non-response as it leads the patient to perceive a lack of genuine care, and the patient may also disengage from the relationship. This finding is consistent with previous research that has identified the perception of distance from the therapist as an unhelpful treatment characteristic [46] and could be interpreted to stem from therapist burnout due to working in overburdened services with inadequate support [58, 73, 86, 89]. Alternatively, therapist disinterest could be due to a lack of therapist knowledge, skill and experience with personality disorder which could result in therapists holding unhelpful attitudes or lack of hope for recovery for people with BPD.

The therapist behaviours discussed in theme seven could be understood as too little emotional input into the therapeutic relationship, whereas the therapist behaviours in theme eight could be interpreted as too much emotional input into the therapeutic relationship, or as countertransference reactions. In the present study, therapists acting on their countertransference may have broken the therapeutic frame by violating the principles of neutrality [21]. In turn, these violations could have compromised the sense of safety in the relationship. People with BPD can experience intense and labile emotions, relational insecurities and impulsive behaviours [3, 12, 79]. When expressed in the treatment setting, these symptoms can be interpreted as resistance to therapy and can create challenges for therapists not to act on their countertransference, thereby contaminating the safety of the holding environment and rupturing the relationship [61, 79, 80, 90, 91]. It is possible that unmanaged countertransference, and lack of therapist support and training, is the underlying cause of the therapist behaviours that patients described as condescending, threatening and shaming. These behaviours leave the patient feeling unsafe in the relationship, and subsequently withdraw and thus non-respond to therapy. It could also be understood that patient vulnerabilities (attachment insecurities, epistemic mistrust and resulting mentalising impairments) can lead to perceptions of threat and mistrust, irrespective of therapist behaviours. The reciprocal interaction between these patient and therapist factors is the most likely reason for the difficulty of the establishment of a safe working alliance.

Domain 4 was created from the insights patients shared regarding what can be altered or emphasised to address the problem of non-response. Domain 4 is foundational to the other Domains; such that if the identified helpful factors were present, the patients would be supported to resolve the barriers to response. A recurrence of the topic of safety emerged again in theme eight where patients asserted that therapy could be more effective if the therapeutic relationship was perceived to be safe. This focus on safety is consistent with a previous meta-synthesis of qualitative studies investigating the experience of treatment for BPD which reported that seven of their 14 included studies also emphasised the importance of safety [46]. Certain therapist characteristics and behaviours were identified as facilitative of the development of safety such as warmth, genuine care and acceptance. Another finding was the recognition that patients perceiving a therapist to be highly attuned and responsive to varying emotions was reported to be supportive of the development of safety. Attunement and responsivity, and the resulting sense of safety, could be explained by effective co-regulation in the room [24, 34, 70].

Theme nine described patients’ wish for therapists to understand and accept that some of the behaviours and emotional responses patients experience can be a result of trauma or context. Patients also noted that it is important for therapists to balance being gentle while pushing for change. This is akin to the foundational psychodynamic method of supportive and expressive techniques [57], the more modern psychodynamic MBT approach of empathic validation vs. challenge [9] and the core component of DBT of balancing the dialectic between acceptance and change [56].

In theme ten, patients emphasised the importance of choice, transparency and collaboration in therapy. Patients felt respected, motivated and empowered to create change when they were invited to have input into their own treatment. Both of these elements promote agency which is essential for recovery [51, 78], and has been previously been highlighted by people with a lived experience of BPD as being important [46]. Patients also discussed how therapy improved when they were given a diagnosis of personality disorder. Being given a diagnosis was described as relieving as it supported self-understanding and fostering hope for recovery. Previous qualitative research has reported that being given a diagnosis of personality disorder was a turning point in the recovery journey because it was facilitative of developing self-insight, feeling validated and obtaining evidence-based treatment [65]. It is notable that approximately half of the patients in this study could not remember getting a formal BPD diagnosis, even though they knew they had the disorder and met current and lifetime criteria. Instead, patients reported recalling being given diagnoses of depression, anxiety, post traumatic stress disorder and bipolar disorders in the past by health care practitioners. Not being communicated a clear and accurate diagnosis of personality disorder may be because of lack of awareness, poor diagnostic practices, clinicians lacking time for thorough diagnostic assessments, or lingering stigma in the mental health community [62, 84]. Regardless of the reasons, the lack of correct communication of diagnosis can create barriers for obtaining correct treatment which may contribute to non-response.

In theme eleven, patients highlighted that for therapy to be considered effective it must focus on reaching achievable goals that result in real life changes. Previous qualitative research has discovered that a common frustration of patients is having misaligned therapeutic goals, where the clinician wants to focus on risk and symptom reduction, while the patient wants to focus on function [45, 64]. An overwhelming amount of longitudinal research reports that people with BPD, even after extensive treatment, remain unable to obtain satisfactory levels of social and occupational function [1, 39, 40, 64]. An unsatisfactory level of focus on practical problem solving and goal attainment may contribute to the difficulties people with BPD have in obtaining satisfactory levels of functioning. A possible solution for this problem could be to incorporate the support of occupational therapists, employment services and student support staff into treatment programs, especially in the early phases.

### Clinical implications

The perspectives of patients reveal that the main factor implicated in non-response to therapy is lack of a sense of safety. The results describe and explain the various manifestations of safety, or lack thereof, and provide a variety of methods for the establishment of safety. Patients declared that external safety must be stabilised before therapy can be effective. While the establishment of external safety is outside of the therapists’ purview, usual practices could be changed such that engagement with community services to stabilise housing, address addiction and ensure the patient is free from active trauma before being referred to clinicians for intensive psychological treatment could significantly reduce non-response. In addition, clinicians and other community services involved could be supported to communicate and take a more collaborative approach. The aspect that is firmly within the scope of the clinician is focusing on the safety of the therapeutic alliance. Therapists could be supported to develop and maintain safe therapeutic relationships with their patients by enhancing their knowledge of personality disorder, the implications of attachment insecurities, the resulting mentalising impairments and countertransference management. Countertransference management can also be supported by viewing ‘challenging’ or ‘resistant’ behaviours expressed by patients as valid adaptive preconscious protective mechanisms that originate in the autonomic nervous system and are influenced by past traumatic experiences, that push them into defensive states [74]. The resultant behaviours can be re-interpreted as patients’ attempt at ensuring their own safety. Another possible method for managing countertransference is for therapists to expand their own ‘windows of tolerance’ so they can tolerate more intense emotional input from patients before activation of their own defensive states occur [34, 74]. Prioritising self-care, wellness and purposeful practice of emotional regulation skills are suggested methods for increasing windows of tolerance and decreasing the likelihood of acting on counter-transferential feelings [74]. The findings also indicate that not being provided with the correct diagnosis of BPD may prohibit obtaining evidence-based treatment for the disorder. Lastly, widening the scope of treatment to focus more on practical life-changing goals in therapy or by including other allied health professionals such as case managers or employment coaches may be helpful for increasing functionality.

### Limitations and future directions

This study had some limitations. The majority of the sample were female, so the perspective of males was poorly represented. The study only encompasses the perspective of patients. Future research could expand this research by investigating non-response from the perspective of families, friends and clinicians. Further research could also attempt to explore whether the phases of treatment response barriers are evident beyond this sample and whether focusing interventions aimed at the phases is effective.

Another limitation of this study is that treatment non-adherence was not directly explored. It is well known that non-compliance is a notable barrier to positive treatment outcomes in medicine and psychiatry [68, 85]. Not following, or ‘sticking to’, the advice that clinicians impart may contribute to non-response. Future research could explore non-compliance as a possible non-response factor. Further, no measure of the severity of BPD symptomology was employed. It would have been useful to assess symptom severity and compare between those with high and low scores. Future research could include measures of symptom severity and social-occupational functioning.

Regarding diagnoses, 16 patients reported having a comorbid diagnosis of PTSD/CPTSD and this could have influenced the high level of importance placed on safety. Regarding treatment, the patients of this study were recruited into a larger longitudinal study after they presented to emergency and then completed a brief stepped-care intervention [38, 42]. Once this intervention was completed patients were referred to local therapists in the community. This prevented the ability to control for therapy type or extent of therapist training.

Lastly, while measures were taken to ensure scientific rigour and credibility of findings, there can be biases inherent to qualitative research. The first author predominantly uses Psychodynamic Theory and Dialectical Behaviour Therapy when treating people with BPD. This may have led to personal biases that could have influenced the study design and the analysis of results. In addition, the lead first author designed the questions, conducted the interviews, and coded the data. It is possible that this invited bias into the analyses of the results. Especially considering that people with BPD can be suggestible in contexts of social desirableness, which could have lead patients to provide answers in alignment with what they think the researcher may be looking for.

## Conclusion

The findings of this research indicate that, for people who have engaged in psychotherapy for BPD, a safe relationship with the therapist is foundational. Not one patient’s reason given to the questions around what makes psychotherapy ineffective was any factor regarding psychotherapeutic modalities, ‘brand names’ or methods. Which is consistent with previous literature that asserts that it is the common factors of evidenced-based therapies that are more effective, not specific therapy modalities per se [11]. The majority of attributions for non-response concerned the safety of the alliance with the therapist. Correspondingly, responses to questions around what makes psychotherapy effective were framed around the therapist and the relationship, and readiness to engage from both patient and therapist. Although this study set out to identify factors related to non-response to psychotherapy, the results demonstrate that development of new therapies may not necessarily be where the focus should lie. It may be that better training and support for therapists working with personality disorder can improve rates of response to therapy for people with BPD. Therefore, shifting the emphasis from psychotherapy development and testing, to patient preparation and therapist training, may be an effective method for addressing non-response to psychotherapy for BPD.

## Supplementary Information

**Additional file 1.** Semi-Structured Interview. Displays the questions asked in the semi-structured interview used to collect the data for this study.

## Data Availability

The dataset generated and analysed during the current study is not publicly available due to the high likelihood that the information contained within it could allow identification of individual patients. However, the interview questions are available as an appendix [see Additional file 1] to allow further interpretation and future replication.
