# Supplementary material for: Patient perspectives on non-response to psychotherapy for borderline personality disorder: a qualitative study
Source: Borderline Personal Disord Emot Dysregul. 2023 Apr 18;10:13. doi: 10.1186/s40479-023-00219-y (PMC10114439; doi:10.1186/s40479-023-00219-y)
Supplement: Supplementary file 1 — Additional file 1. Semi-Structured Interview. Displays the questions asked in the semi-structured interview used to collect the data for this study. [file 40479_2023_219_MOESM1_ESM.docx]

**Semi-Structured Interview**

Before we start, I just want to check that we are using the same definition of psychotherapy – when I say psychotherapy I mean talk therapy, with a psychologist or counsellor. Are we on the same page here? Does that match your definition?

I would like to start by asking you about your perspectives on response and non-response to psychotherapy *(for borderline personality disorder).*

So, we know that many people *(with BPD)* respond well to psychotherapy.

*(Clarify ‘respond’ if necessary)*

We also know that around one third don’t respond.

Why do you think that is?

1. Could you define what response to psychotherapy for (borderline personality disorder) means to you? As in, how would you know if it was working?
2. What aspects of psychotherapy are effective and why?
3. What aspects of psychotherapy are ineffective and why?
4. From your perspective what makes a therapist effective?
5. From your perspective what makes a therapist ineffective?
6. Do you feel that you have responded to psychotherapy?

If participant feels they have improved/recovered:

1. Why do you think you were able to respond to psychotherapy?
2. What exactly has helped you respond to psychotherapy?
3. What hindered your ability to respond to psychotherapy?
4. What needed to change for you to be able to respond?
5. Did you experience a turning point or a pivotal moment during your psychotherapy?

*Prompts:*

(If yes) Can you describe this to me?

(If yes) What lead you to this turning point?”

If participant feels they have NOT improved/recovered:

1. Why do you think you have been unable to respond to psychotherapy?
2. Can you think of anything that has acted as a barrier to your ability to respond to psychotherapy?
3. What might need to change for you to be able to respond to psychotherapy?
4. How could your psychotherapy be modified to help you respond?
5. Can you think of anything that has helped you respond so far?
6. Do you think you will be able to respond in the future?

*Prompt:* Can you say a little more about this?
